# Effects of the interaction between PTSD and ADHD symptoms on the level of reporting psychotic-like experiences: findings from a non-clinical population

**DOI:** 10.3389/fpsyt.2023.1232606

**Published:** 2023-10-06

**Authors:** Hanna Gelner, Julia Karska, Łukasz Gawęda, Jerzy Samochowiec, Błażej Misiak

**Affiliations:** ^1^Experimental Psychopathology Lab, Institute of Psychology, Polish Academy of Sciences, Warsaw, Poland; ^2^Department of Psychiatry, Wroclaw Medical University, Wroclaw, Poland; ^3^Department of Psychiatry, Pomeranian Medical University, Szczecin, Poland

**Keywords:** ADHD, PTSD, psychosis, comorbidity, early intervention

## Abstract

**Objective:**

Psychotic-like experiences (PLEs) are increasingly being recognized as subclinical phenomena that might predict the development of various mental disorders that are not limited to the psychosis spectrum. Accumulating evidence suggests that attention-deficit/hyperactivity disorder (ADHD) and post-traumatic stress disorder (PTSD) are highly comorbid mental disorders. However, their interactive effect on the occurrence of PLEs has not been investigated so far. Therefore, in the present study we aimed to investigate the effect of interaction between ADHD and PTSD symptoms on the level of psychotic-like experiences (PLEs) in the non-clinical sample.

**Methods:**

The study included 3,000 individuals aged 18–35 years with a negative history of psychiatric treatment. The symptoms of ADHD and PTSD were assessed using self-reports.

**Results:**

There was a significant association of the interaction between ADHD and PTSD with the level of reporting PLEs. This association remained significant after adjustment for age, gender, the level of education, the current vocational situation, lifetime history of problematic substance use, and depressive symptoms. Post-hoc tests demonstrated significantly higher levels of reporting PLEs in participants with positive screening for both ADHD and PTSD compared to other subgroups of participants. Also, individuals with positive screening for one vulnerability (either ADHD or PTSD) reported significantly higher levels of reporting PLEs compared to those with a negative screening for ADHD and PTSD. In turn, no significant differences between individuals reporting one vulnerability, i.e., between those with positive screening for ADHD and those with positive screening for PTSD, were observed.

**Conclusion:**

Findings from the present study imply that both PTSD and ADHD symptoms the interaction effect on the level of reporting PLEs that might be of importance for early intervention strategies. However, observed associations require replication in clinical samples.

## 1. Introduction

Psychotic-like experiences (PLEs) represent frequent psychopathological phenomena with the median annual prevalence estimated at 7.2% according to a systematic review and meta-analysis (1). Most commonly, PLEs are defined as subclinical phenomena that develop in the absence of underlying illness in terms of international diagnostic systems (2–4). It has been shown that the emergence of PLEs is associated with increased risk of developing mental disorders that are not limited to psychosis spectrum, suicidal behavior, multimorbidity, and poor functioning (5, 6). Although a great progress toward understanding the mechanisms contributing to the development of PLEs has been made in recent years, certain aspects remain unclear.

One of the most crucial mechanisms that might account for the appearance of psychosis spectrum symptoms are alterations in the process of neurodevelopment (7, 8). It has been reported that psychotic symptoms often occur in vulnerable populations affected by common neurodevelopmental disorders. Among them, several studies have focused on attention-deficit/hyperactivity disorder (ADHD) that might persist until adulthood, and affect even 2.5% of adults (9, 10). It has been demonstrated that ADHD diagnosed in childhood is associated with a fivefold higher risk of psychotic disorder in adulthood (11). Moreover, ADHD is highly comorbid with other mental disorders, including, i.e., autism spectrum disorder (12), mood disorders (13, 14), substance use disorders (15, 16).

Exposure to traumatic events is a well-known trigger of various psychiatric disorders, including post-traumatic stress disorder (PTSD) with prevalence rates estimated at 5.6% of trauma-exposed individuals (17). The association between PTSD and psychotic symptoms has been established by several studies and summarized in the ICD-11 for both PTSD and complex PTSD. Moreover, certain risk factors for psychotic symptoms secondary to a diagnosis of PTSD have been reported. These include trauma-related characteristics (childhood trauma and war trauma), refugee and ethnic minority status, comorbid mood disorders and substance use (18). PLEs secondary to PTSD usually include persecutory ideation and auditory or visual hallucination-like experiences (19, 20). Moreover, there is evidence that psychotherapeutic interventions based on cognitive behavioral therapy (CBT) and eye movement and desensitization and reprocessing therapy (EMDR) might be effective for people with PTSD comorbid with other mental disorders and psychotic symptoms (18).

Accumulating evidence indicates that ADHD and PTSD tend to co-occur and might be causally associated (21). Indeed, the most recent Mendelian randomization study demonstrated that ADHD genetic liability increases the risk of PTSD (22). Authors of this study found that risk-taking behaviors, household income, and educational attainment might only partially mediate this association. Specific neurobiological mechanisms underlying the association between ADHD and PTSD are yet to be determined. Nevertheless, it has been found that prenatal nicotine exposure leads to the development of ADHD-like behaviors together with impairments of fear extinction learning in mice (23, 24). The later one might also occur in people with PTSD (25). There are also studies showing beneficial effects of methylphenidate on fear extinction and PTSD symptoms (26, 27). Also, a number of risk factors for comorbid ADHD and PTSD have been reported including the effect of low intelligence quotient, a history of traumatic experiences in parents, alcohol use disorders and familial transmission of this comorbidity (28–30). It has been shown that a diagnosis of PTSD in individuals with ADHD is associated with a higher risk of psychiatric hospitalization, school impairment, worse social and cognitive performance as well as a higher likelihood of mood, conduct disorder, and anxiety disorders (29, 31).

To date, none of previous studies have investigated the interactive effects of PTSD and ADHD symptoms on the occurrence of PLEs. In the present study, we hypothesized that PTSD and ADHD symptoms exert interaction effects on the level of reporting PLEs. Our aim was to explore the interaction between PTSD and ADHD symptoms in a non-clinical population of young adults.

## 2. Methods

### 2.1. Recruitment procedures

The total sample included 3,000 individuals recruited through social media and the survey website. Recruitment was conducted via an online survey, with the advertisement targeted at individuals aged 18–35 years, without a history of psychiatric treatment. The findings reported in the present article represent a part of a bigger project investigating epigenetic mechanisms of psychosis proneness. At the screening level, the survey included only email addresses and telephone numbers for participants willing to participate in further stages of the project. Additional personal data were not collected at this stage of the project. Some findings from this study were published previously (32–34). The study was approved by the Ethics Committees at the Institute of Psychology (Polish Academy of Sciences in Warsaw, Poland, approval number: 16/VII/2022), Wroclaw Medical University (Wroclaw, Poland, approval number: 129/2022) and Pomeranian Medical University (Szczecin, Poland, approval number: KB-006/25/2022).

### 2.2. Measures

#### 2.2.1. PLEs

The occurrence of PLEs was assessed using a 16-item self-report referring to the preceding month. Participants were asked to assess the frequency of PLEs on a 4-point scale (1 – “never”; 2 – “sometimes”; 3 – “often” and 4 – “almost always”), taking into consideration the experiences that had not been related to substance use. Specific items were selected from three questionnaires, including: (1) the Revised Hallucination Scale (35–37): “I hear voice speaking my thoughts aloud,” „I hear people call my name and find that nobody has done so” and “I see shadows and shapes when there is nothing there”, (2) the Revised Green et al., Paranoid Thoughts Scale (38): “I spent time thinking about friends gossiping about me,” “People wanted me to feel threatened, so they stared at me,” “I was convinced there was a conspiracy about me,” “I was distressed by being persecuted” and “People have been dropping hints for me”, and (3) the Prodromal Questionnaire (39): “When I look at a person, or look at myself in a mirror, I have seen the face change right before my eyes,” “I have heard things other people cannot hear like voices of people whispering or talking,” “I often feel that other have it in for me,” “I have seen things that other people apparently cannot see,” “I have had the sense that some person or force is around me, even though I could not see anyone,” “I sometimes see special meanings in advertisements, shop windows, or in the way things are arranged around me,” “I sometimes smell or taste things that other people cannot smell or taste” and “I often seem to live through events exactly as they happened before.” We decided to use items from various questionnaires as existing tools often record the presence of various experiences including hallucination-like experiences, delusion-like experiences, negative, and depressive symptoms (40). The total score of this questionnaire ranges between 16 and 64 points, with higher scores referring to the greater number and frequency of PLEs. The Cronbach’s alpha of the questionnaire in the present study was 0.784.

### 2.3. The screening for ADHD

The Adult ADHD Self-Report Scale (ASRS) was administered to screen for ADHD. It includes 9 items measuring inattention and 9 items measuring impulsivity/hyperactivity over the period of the preceding 6 months (41). Each item is based on a 5-point scale (from 0 – “never” to 4 – “very often”). The total ASRS score ranges between 0 and 72. The first six items of the ASRS were found to show the highest utility in screening for ADHD. It has been found that individuals scoring positively on at least four of these items should be further assessed for a diagnosis of ADHD (41). Positively scored items include those with the symptom frequency reported to be at least 2 – “sometimes” (items 1–3) or 3 – often (items 4–6). Following this threshold, participants were divided into those with positive screening for ADHD and those with negative screening for ADHD. The Cronbach’s alpha of the ASRS was 0.819 in the present study.

### 2.4. The screening for PTSD

Participants were screened for a diagnosis of PTSD using three questions from the Mini-International Neuropsychiatric (M.I.N.I.) (42): 1) “Have you ever experienced, witnessed or faced an extremely traumatic event, during which people have died or you and/or other persons been in danger of being killed, were severely injured or were harmed with regard to their physical integrity? Examples of traumatic events: severe accident, physical assault, rape, terrorism, hostage-taking, kidnapping, fire, discovering a dead body, sudden death of a significant other, war, natural disasters.”; 2) “Do you often think of this event in a distressing way, do you dream about it or do you frequently have the impression of re-experiencing it?” 3) “Since the occurrence of this event, have you had a tendency to avoid everything that could remind you of the event?.” Participants were classified as screened positive in case of positive responses to all questions.

### 2.5. Depressive symptoms

Depressive symptoms were measured using the Patient Health Questionnaire-9 (PHQ-9) (43). It is a 9-item questionnaire that measures the level of depressive symptoms over the preceding two weeks. Each item of the PHQ-9 is based on a 4-point scale that records the frequency of depressive symptoms (from 0 – “not at all” to 3 – “nearly every day”). The total PHQ-9 score ranges between 0 and 27 with higher scores corresponding with greater severity of depressive symptoms. The Cronbach’s alpha for the PHQ-9 was 0.763 in our sample.

### 2.6. Other measures

Participants were also asked about age, gender, the level of education, as well a family history of schizophrenia and mood disorders. The level of education was categorized as follows: (1) primary (graduation from primary school only), (2) vocational (gives preparation for professional employment, based on education in basic vocational schools, basic schools or other equivalent schools, or study of crafts); (3) secondary (graduation from a general or vocational secondary school), (4) incomplete higher (a person studied at a university, but did not obtain a diploma), and (5) higher (obtaining a bachelor’s, engineer’s, master’s, or equivalent degree; this category also includes the PhD degree). Current vocational situation [(unemployed, i.e., lack of employment), student (in the course of studies, i.e., first, second or third degree) without full-time job), employed (full-time job), rent (on social support/ security benefits)]. The lifetime problematic substance use was assessed using the screening question from the M.I.N.I. (“Have you ever taken any of these drugs, on one or more occasions, to get better, to get a good mood, or to change your current mood?”; the list of various substances has been provided) (42). Positive response to this question was coded as “problematic substance use.”

### 2.7. Data analysis

The analysis of variance (ANOVA) was used to test the association of positive screening for PTSD and ADHD (the independent variables) with the level of reporting PLEs (the dependent variable). In the first ANOVA model, results of screening for PTSD and ADHD together with the interaction term (PTSD × ADHD screening results) were included as the only independent variables. The interaction term (PTSD × ADHD screening results) resulted in creating four groups: (1) individuals who screened positive for ADHD, but not for PTSD (the ADHD(+), PTSD(−) group), (2) individuals who screened positive for PTSD, but not for ADHD (the ADHD(−), PTSD(+) group), (3) individuals who screened positive for both ADHD and PTSD (the ADHD(+), PTSD(+) group), and (4) individuals who screened negative for ADHD and PTSD (the ADHD(−), PTSD(−) group). Next, sociodemographic characteristics, including age, gender, the level of education (higher or incomplete higher vs. other) and the current vocational situation (employed or student vs. other) were added as covariates. Finally, the lifetime history of problematic substance use and the PHQ-9 score were added to the ANOVA model as covariates. Post-hoc comparisons were performed using the Games-Howell test. The results of data analysis were interpreted as significant in case of *p* < 0.05. All analyses were carried out using the SPSS software (version 28).

## 3. Results

The majority of participants were females (58.3%), individuals with a higher level of education (40.7%), students (51.9%) and people reporting being married or informal relationship (66.6%) (see Table 1 for sample characteristics). Family history of schizophrenia and mood disorders among first- or second-degree relatives was reported by 4.9 and 19.9% of the sample, respectively. A history of problematic substance use was declared by 37.2% of participants. The analysis of data from the questionnaires screening for ADHD and PTSD revealed the following findings: 1) 18.4% were screened positive for ADHD, but not for PTSD (the ADHD(+), PTSD(−) group); 2) 3.7% were screened positive for PTSD, but not for ADHD (the ADHD(−), PTSD(+) group) and 3) 3.7% were screened positive for both ADHD and PTSD (the ADHD(+), PTSD(+) group). Specific subgroups of participants differed significantly with respect to age, gender, education, rates of family history of mood disorders, self-reported lifetime problematic substance use, the level of reporting PLEs, and the PHQ-9 score.

**Table 1:** Descriptive characteristics of the sample.

	The whole sample	ADHD(−), PTSD(−) [*n* = 2,222]	ADHD(−), PTSD(+) [*n* = 111]	ADHD(+), PTSD(−) [*n* = 553]	ADHD(+), PTSD(+) [*n* = 111]	*p*
Age	25.4 ± 4.9	25.6 ± 4.9	26.4 ± 5.1	24.8 ± 4.9	24.4 ± 4.7	< 0.001
Gender, males	1,250 (41.7)	990 (44.6)	41 (36.9)	196 (35.4)	23 (20.7)	< 0.001
Education						
Primary	101 (3.4)	67 (3.0)	3 (2.7)	22 (4.0)	9 (8.1)	
Vocational	46 (1.5)	33 (1.4)	0 (0)	9 (1.6)	4 (3.6)	0.003
Secondary	911 (30.4)	657 (29.6)	42 (37.9)	172 (31.1)	40 (36.0)	
Incomplete higher	720 (24.0)	519 (23.4)	26 (23.4)	152 (27.5)	23 (20.7)	
Higher	1,222 (40.7)	949 (42.6)	40 (36.0)	198 (35.8)	35 (31.6)	
Vocational situation						
Unemployed	113 (3.8)	89 (4.0)	5 (4.5)	15 (2.7)	5 (4.6)	
Student	1,557 (51.9)	1,133 (51.0)	54 (48.7)	315 (57.0)	55 (49.6)	0.180
Employed	1,300 (43.3)	984 (44.1)	49 (44.1)	216 (39.0)	51 (45.9)	
Pension	30 (1.0)	19 (0.9)	3 (2.7)	7 (1.3)	1 (0.9)	
Married or informal relationship	1999 (66.6)	1,490 (67.1)	80 (72.1)	358 (64.7)	71 (64.0)	0.386
Family history of schizophrenia	146 (4.9)	97 (4.4)	7 (6.3)	34 (6.1)	8 (7.2)	0.171
Family history of mood disorder	597 (19.9)	390 (17.6)	35 (31.5)	131 (23.7)	41 (36.9)	< 0.001
Lifetime problematic substance use	1,115 (37.2)	757 (34.1)	43 (38.7)	255 (46.1)	60 (54.0)	< 0.001
ASRS	30.2 ± 10.3	27.0 ± 8.5	30.0 ± 8.4	40.2 ± 8.7	44.1 ± 9.4	< 0.001
PLEs	22.5 ± 4.9	21.7 ± 4.1	24.1 ± 5.8	24.0 ± 5.2	28.6 ± 8.9	< 0.001
HLEs	8.9 ± 2.1	8.7 ± 1.8	9.5 ± 2.7	9.3 ± 2.1	10.9 ± 3.9	< 0.001
DLEs	13.6 ± 3.4	13.0 ± 2.9	14.6 ± 3.8	14.7 ± 3.7	17.7 ± 5.9	< 0.001
PHQ-9	9.4 ± 5.2	8.4 ± 4.6	11.2 ± 5.2	12.0 ± 5.2	15.5 ± 6.4	< 0.001

Data presented as mean ± SD or *n* (%).

ASRS, the Adult ADHD Self-Report Scale; DLEs, delusion-like experiences; HLEs, hallucination-like experiences; PHQ-9, the Patient Health Questionnaire-9; PLEs, psychotic-like experiences.

**Figure 1:**
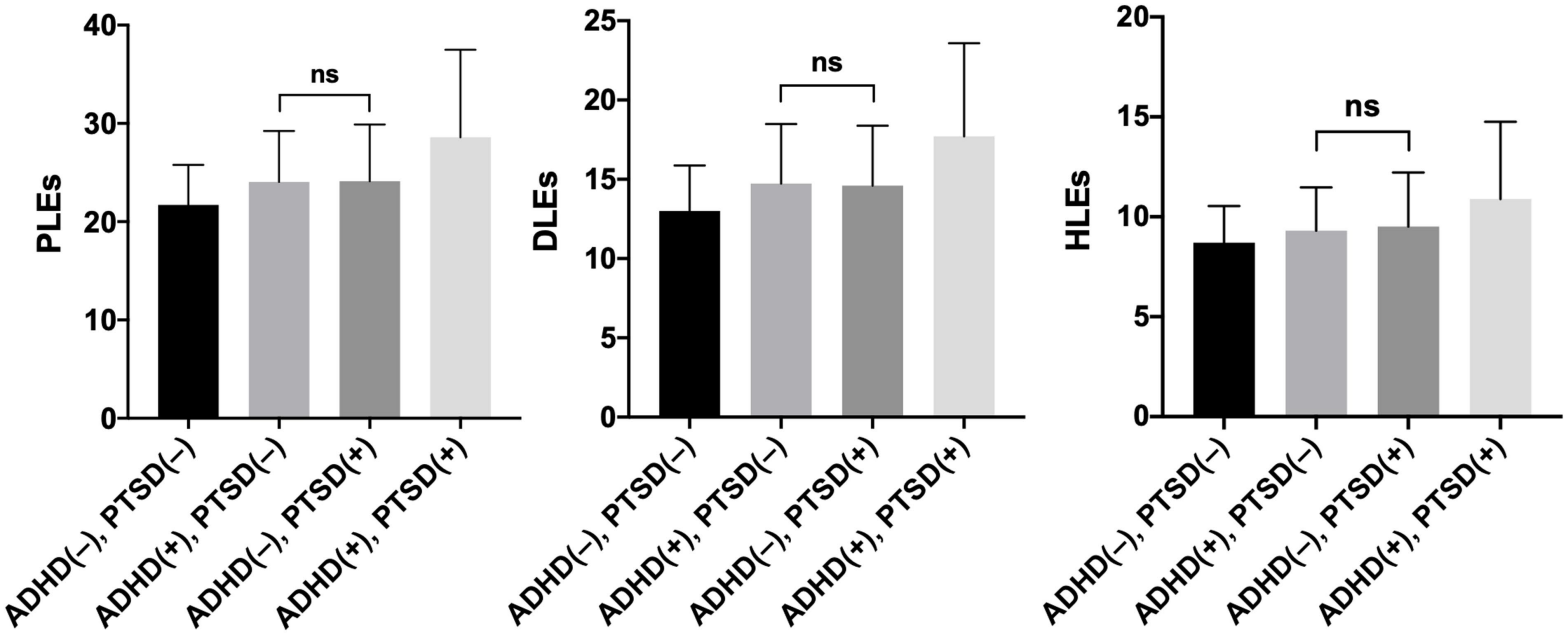
The level of psychotic-like experiences (PLEs), delusion-like experiences (DLEs) and hallucination-like experiences (HLEs) in participants with various combinations of ADHD and PTSD. Mean values and corresponding 95%CI (error bars) are shown. Post-hoc tests revealed significant differences of all bivariate comparisons, except for the comparison indicated as “ns”.

The ANOVA demonstrated significant effects of screening for PTSD and ADHD as well as the ADHD × PTSD interaction on the level of reporting PLEs in general, including delusion-like experiences (DLEs) and hallucination-like experiences (HLEs), even after adding potential covariates to the model (Table 2). The models with all potential covariates explained 26.1, 27.0 and 11.9% of the variance in reporting PLEs, DLEs and HLEs, respectively (by means of the adjusted R^2^). Post-hoc tests demonstrated that the ADHD(+), PTSD(+) individuals had significantly higher levels of reporting PLEs, DLEs and HLEs compared to the ADHD(+), PTSD(−) individuals as well as the ADHD(−), PTSD(+) individuals (Figure 1, Table 3). The ADHD(+), PTSD(−) individuals as well as the ADHD(−), PTSD(+) individuals did not differ significantly with respect to the level of reporting PLEs, DLEs, and HLEs. However, both groups of participants had significantly higher levels of reporting PLEs, DLEs, and HLEs compared to the ADHD(−), PTSD(−) individuals.

**Table 2:** The association of positive screening for ADHD and PTSD with PLEs after controlling for the effects of potential confounding factors.

Model	Independent variable	Dependent variable		
		PLEs	DLEs	HLEs
Model 1 (ADHD + PTSD)	ADHD	*F* = 158.342, p < 0.001	*F* = 156.648, *p* < 0.001	*F* = 74.281, *p* < 0.001
	PTSD	*F* = 132.799, p < 0.001	*F* = 120.507, p < 0.001	*F* = 75.018, p < 0.001
	ADHD × PTSD	*F* = 16.329, p < 0.001	*F* = 12.461, p < 0.001	*F* = 12.558, p < 0.001
Model 2 (ADHD + PTSD + sociodemographic characteristics)	ADHD	*F* = 153.580, p < 0.001	*F* = 149.833, p < 0.001	*F* = 74.112, p < 0.001
	PTSD	*F* = 129.647, p < 0.001	*F* = 115.684 p < 0.001	*F* = 75.365 p < 0.001
	ADHD × PTSD	*F* = 15.672, p < 0.001	*F* = 11.589, p < 0.001	*F* = 12.570, p < 0.001
	Age	*F* = 8.012, *p* = 0.005	*F* = 7.084, *p* = 0.008	*F* = 4.741, *p* = 0.030
	Gender	*F* = 4.171, *p* = 0.041	*F* = 1.350, *p* = 0.245	*F* = 7.636, *p* = 0.006
	Education	*F* = 38.976 p < 0.001	*F* = 55.627, p < 0.001	*F* = 5.318, *p* = 0.021
	Vocational status	*F* = 2.151, *p* = 0.143	*F* = 1.361, *p* = 0.243	*F* = 2.138, *p* = 0.144
Model 3 (ADHD + PTSD + sociodemographic characteristics + depression + substance use)	ADHD	*F* = 52.723, p < 0.001	*F* = 49.430, p < 0.001	*F* = 25.376, p < 0.001
	PTSD	*F* = 65.350, p < 0.001	*F* = 54.156, p < 0.001	*F* = 39.746, p < 0.001
	ADHD × PTSD	*F* = 12.808, p < 0.001	*F* = 8.826, *p* = 0.003	*F* = 10.354, *p* = 0.001
	Age	*F* = 10.063, p < 0.001	*F* = 8.984, p = 0.003	*F* = 5.343, p = 0.021
	Gender	*F* = 13.752, p < 0.001	*F* = 7.846, p = 0.005	*F* = 14.036, p < 0.001
	Education	*F* = 32.333, p < 0.001	*F* = 48.866, p < 0.001	*F* = 3.022, *p* = 0.082
	Vocational status	*F* = 1.081, *p* = 0.299	*F* = 0.492, *p* = 0.483	*F* = 1.380, *p* = 0.240
	Depression	*F* = 540.563, p < 0.001	*F* = 572.34, p < 0.001	*F* = 196.916, p < 0.001
	Substance use	*F* = 8.755, p = 0.003	*F* = 5.811, *p* = 0.016	*F* = 7.443 p = 0.006

ADHD, attention-deficit/hyperactivity disorder; DLEs, delusion-like experiences; HLEs, hallucination-like experiences; PLEs, psychotic-like experiences; PTSD, post-traumatic stress disorder.

**Table 3:** Results of the post-hoc comparisons.

Comparison	PLEs			DLEs			HLEs		
	Mean difference	SE	*p*	Mean difference	SE	*p*	Mean difference	SE	*p*
ADHD(−), PTSD(−) vs. ADHD(−), PTSD(+)	−2.42	0.55	< 0.001	−1.61	0.36	< 0.001	−0.81	0.26	0.012
ADHD(−), PTSD(−) vs. ADHD(+), PTSD(−)	−2.34	0.24	< 0.001	−1.74	0.17	< 0.001	−0.61	0.10	< 0.001
ADHD(−), PTSD(−) vs. ADHD(+), PTSD(+)	−6.89	0.85	< 0.001	−4.71	0.56	< 0.001	−2.19	0.37	< 0.001
ADHD(−), PTSD(+) vs. ADHD(+), PTSD(−)	0.07	0.59	0.999	−0.13	0.39	0.987	0.20	0.27	0.877
ADHD(−), PTSD(+) vs. ADHD(+), PTSD(+)	−4.48	1.01	< 0.001	−3.10	0.66	< 0.001	−1.40	0.45	0.012
ADHD(+), PTSD(−) vs. ADHD(+), PTSD(+)	−4.55	0.87	< 0.001	−2.97	0.58	< 0.001	−1.58	0.38	< 0.001

ADHD, attention-deficit/hyperactivity disorder; DLEs, delusion-like experiences; HLEs, hallucination-like experiences; PTSD, post-traumatic stress disorder.

## 4. Discussion

The current study examined the effect of the interaction between PTSD and ADHD symptoms on the level of reporting PLEs in a non-clinical sample. We found that both groups of symptoms show an interaction effect on the level of reporting PLEs in this population. Hence, our results add to the literature showing that PTSD (18) as well as ADHD (11, 44) might be associated with reporting PLEs (11, 18, 29, 31, 44). It should be noted that our study was based on a non-clinical sample, and thus we refer to the level of reporting PLEs. However, some studies have also suggested that PLEs increase the risk of subsequent psychotic disorders (4). Given the fact that we investigated only a simple interaction effect of two potential risk factors on the level of reporting PLEs, we were not able to provide mechanisms of how these factors impact the occurrence of PLEs. More specifically, our results suggest that individuals who have ADHD and PTSD symptoms show significantly higher levels of reporting PLEs compared to the group of individuals without ADHD and PTSD symptoms. Importantly, our results were similar for both types of PLEs, i.e., DLEs and HLEs. In line with prior studies (11, 45), our results can be interpreted as showing an interaction between PTSD and ADHD symptoms on the occurrence of PLEs. Our study suggests a significant interaction between PTSD, ADHD and PLEs. According to our results, we recommend that this interaction should be further investigated.

When interpreting our results, it should be noted that our sample can be described as a well-functioning social group. We assessed both PTSD and ADHD symptoms as a continuum in a non-clinical sample. Hence, more severe states within narrow clinical criteria for respective disorders were not represented in the study. At the same time, several studies indicate the role of exposure to trauma in shaping the risk of psychosis, both in clinical (46) and non-clinical studies (47). In a clinical context, comorbidity of ADHD and PTSD has already been reported in prior studies showing the impact on general functioning. For instance, results of the study by Antshel et al. (29) suggested that the co-occurrence of PTSD and ADHD in the adult group leads to a greater degree of clinical severity in terms of co-occurring psychiatric disorders and psychosocial functioning. Biederman et al. (31) demonstrated similar results showing that PTSD comorbidity in individuals with a diagnosis of ADHD leads to greater clinical severity in terms of psychiatric comorbidity and psychosocial dysfunction in adolescents. Also, it should be noted that people with ADHD are more likely to be traumatized due to their impulsivity and risky behavior (48). More specifically ADHD is associated with deficits in cognitive and emotion regulation (e.g., high impulsivity, impaired inhibitory ability) which potentially increases the risk of being exposed to the experience of behavior perceived as “socially problematic” (48). For example, during developmental periods, and later, these individuals are more likely to experience physical trauma, lower performance at work/school, more interpersonal conflict, or exposure to risky behaviors (e.g., gambling, substance abuse, or unsafe sexual behavior) that can lead to the experience of trauma or the experience of violence (48, 49).

Referring to the literature, previous studies that have examined the interaction between the occurrence of PTSD and/or ADHD and substance abuse have noted that certain risk factors for the onset of psychotic symptoms secondary to a diagnosis of PTSD and ADHD are present, including problematic substance use (28). In addition, a strong association has been shown between problematic substance use and positive screening results for PLEs (50, 51), which is also consistent with the results obtained in our study. Moreover, other studies report that in the group of individuals with ADHD, alcohol abuse can be related to a higher frequency of more severe PTSD symptoms (28). Individuals with ADHD diagnosis appear to be more likely to exposure to risky behaviors (e.g., substance abuse) which can be caused, for example, by impaired response inhibition, high impulsivity, or sensation seeking (48, 49, 52). On the other hand, problematic substance use can also be associated with prior experience of trauma (53–55). Indeed, previous studies have indicated high rates of co-occurrence of PTSD and substance abuse. Substance abuse may be, for instance, an attempt to avoid traumatic memories (including the associated distress experienced), but on the other hand, through substance abuse, individuals may expose themselves to traumatic experiences and events (55). Therefore, problematic substance use may largely increase the chance of occurrence of PLEs in the future, especially for those diagnosed with ADHD and/or PTSD as overlapping factors. We suggest that this should be explored in more detail by future studies.

There are important limitations of the present study that need to be discussed. First, the study was based on a snowball sampling method that might be characterized by certain shortcomings related to the accuracy of assessments (56). Second, the use of a non-clinical sample with implementation of screening tools does not allow to discuss valid diagnostic constructs according to international classifications of mental disorders. This might also underlie high rates of problematic substance use found in the present sample. Third, the use of self-reports might be characterized by a recall bias. However, certain constructs assessed using self-reports in the present study might still hold some validity. For instance, there is evidence that self-reported PLEs, even if they are found to serve as false-positive observations upon clinical assessment, may still predict the development of mental disorders, including psychosis (5, 57, 58). Fourth, our sample was limited to individuals aged 18–35 years, and thus generalization of findings should be approached with caution. Finally, causal associations cannot be made as we used a cross-sectional design.

To conclude, the current study investigated the effect of the interaction between PTSD and ADHD symptoms on the risk of PLEs in a non-clinical sample. The results from our study show that both PTSD and ADHD symptoms might have an interaction effect on the level of reporting PLEs. Our results also suggest that individuals who have both PTSD and ADHD symptoms have higher levels of PLEs compared to individuals without these symptoms. This observation highlights the importance of investigating the interaction between PTSD and ADHD on the occurrence of PLEs. Future studies need to investigate our findings in longitudinal cohorts and clinical populations in order to provide specific recommendations for clinical practice. In case of replication within clinical cohorts, findings from the present study would raise awareness about the importance of PTSD and ADHD comorbidity in clinical practice. Also, the findings might provide grounds for investigating the most effective therapeutic interventions in this clinical population.

## Data availability statement

The datasets presented in this study can be found in online repositories. The names of the repository/repositories and accession number(s) can be found at: https://osf.io/8ju6b.

## Ethics statement

The studies involving humans were approved by the study was approved by the Ethics Committees at the Institute of Psychology (Polish Academy of Sciences in Warsaw, Poland, approval number: 16/VII/2022), Wroclaw Medical University (Wroclaw, Poland, approval number: 129/2022) and Pomeranian Medical University (Szczecin, Poland, approval number: KB-006/25/2022). The studies were conducted in accordance with the local legislation and institutional requirements. The participants provided their written informed consent to participate in this study.

## Author contributions

HG and JK: writing – original draft. ŁG and JS: writing – review and editing and conceptualization. BM: conceptualization, funding acquisition, formal analysis, writing – original draft, and writing – review and editing. All authors contributed to the article and approved the submitted version.

## References

[1] Linscott RJ van Os J. An updated and conservative systematic review and meta-analysis of epidemiological evidence on psychotic experiences in children and adults: on the pathway from proneness to persistence to dimensional expression across mental disorders. Psychol Med. (2013) 43:1133–49. doi: 10.1017/S0033291712001626, PMID: 22850401

[2] Kelleher I Keeley H Corcoran P Lynch F Fitzpatrick C Devlin N. Clinicopathological significance of psychotic experiences in non-psychotic young people: evidence from four population-based studies. Br J Psychiatry. (2012) 201:26–32. doi: 10.1192/bjp.bp.111.101543, PMID: 22500011

[3] Linscott RJ van Os J. Systematic reviews of categorical versus continuum models in psychosis: evidence for discontinuous subpopulations underlying a psychometric continuum. Implications for DSM-V, DSM-VI, and DSM-VII. Annu Rev Clin Psychol. (2010) 6:391–419. doi: 10.1146/annurev.clinpsy.032408.153506, PMID: 20192792

[4] Kelleher I Cannon M. Psychotic-like experiences in the general population: characterizing a high-risk group for psychosis. Psychol Med. (2011) 41:1–6. doi: 10.1017/S0033291710001005, PMID: 20624328

[5] Kaymaz N Drukker M Lieb R Wittchen HU Werbeloff N Weiser M. Do subthreshold psychotic experiences predict clinical outcomes in unselected non-help-seeking population-based samples? A systematic review and meta-analysis, enriched with new results. Psychol Med. (2012) 42:2239–53. doi: 10.1017/S0033291711002911, PMID: 22260930

[6] Kelleher I Devlin N Wigman JT Kehoe A Murtagh A Fitzpatrick C. Psychotic experiences in a mental health clinic sample: implications for suicidality, multimorbidity and functioning. Psychol Med. (2014) 44:1615–24. doi: 10.1017/S0033291713002122, PMID: 24025687

[7] Khandaker GM Stochl J Zammit S Lewis G Jones PB. A population-based longitudinal study of childhood neurodevelopmental disorders, IQ and subsequent risk of psychotic experiences in adolescence. Psychol Med. (2014) 44:3229–38. doi: 10.1017/S0033291714000750, PMID: 25066026 PMC4180723

[8] Murray RM Lewis SW. Is schizophrenia a neurodevelopmental disorder? Br Med J (Clin Res Ed). (1987) 295:681–2. doi: 10.1136/bmj.295.6600.681, PMID: 3117295 PMC1247717

[9] Polanczyk G de Lima MS Horta BL Biederman J Rohde LA. The worldwide prevalence of ADHD: a systematic review and metaregression analysis. Am J Psychiatry. (2007) 164:942–8. doi: 10.1176/ajp.2007.164.6.942, PMID: 17541055

[10] Simon V Czobor P Balint S Meszaros A Bitter I. Prevalence and correlates of adult attention-deficit hyperactivity disorder: meta-analysis. Br J Psychiatry. (2009) 194:204–11. doi: 10.1192/bjp.bp.107.048827, PMID: 19252145

[11] Nourredine M Gering A Fourneret P Rolland B Falissard B Cucherat M. Association of Attention-Deficit/hyperactivity disorder in childhood and adolescence with the risk of subsequent psychotic disorder: a systematic review and Meta-analysis. JAMA Psychiatry. (2021) 78:519–29. doi: 10.1001/jamapsychiatry.2020.4799, PMID: 33625499 PMC7905700

[12] Rong Y Yang C-J Jin Y Wang Y. Prevalence of attention-deficit/hyperactivity disorder in individuals with autism spectrum disorder: a meta-analysis. Res Autism Spectr Disord. (2021) 83:101759. doi: 10.1016/j.rasd.2021.101759

[13] Bartoli F Callovini T Cavaleri D Cioni RM Bachi B Calabrese A. Clinical correlates of comorbid attention deficit hyperactivity disorder in adults suffering from bipolar disorder: a meta-analysis. Aust N Z J Psychiatry. (2023) 57:34–48. doi: 10.1177/00048674221106669, PMID: 35786010

[14] Meinzer MC Pettit JW Viswesvaran C. The co-occurrence of attention-deficit/hyperactivity disorder and unipolar depression in children and adolescents: a meta-analytic review. Clin Psychol Rev. (2014) 34:595–607. doi: 10.1016/j.cpr.2014.10.002, PMID: 25455624

[15] Bartoli F Callovini T Calabrese A Cioni RM Riboldi I Crocamo C. Disentangling the association between ADHD and alcohol use disorder in individuals suffering from bipolar disorder: a systematic review and Meta-analysis. Brain Sci. (2021) 12:12010038. doi: 10.3390/brainsci12010038, PMID: 35053783 PMC8773515

[16] Hartman CA Larsson H Vos M Bellato A Libutzki B Solberg BS. Anxiety, mood, and substance use disorders in adult men and women with and without attention-deficit/hyperactivity disorder: a substantive and methodological overview. Neurosci Biobehav Rev. (2023) 151:105209. doi: 10.1016/j.neubiorev.2023.105209 37149075

[17] Koenen KC Ratanatharathorn A Ng L McLaughlin KA Bromet EJ Stein DJ. Posttraumatic stress disorder in the world mental health surveys. Psychol Med. (2017) 47:2260–74. doi: 10.1017/S0033291717000708, PMID: 28385165 PMC6034513

[18] Compean E Hamner M. Posttraumatic stress disorder with secondary psychotic features (PTSD-SP): diagnostic and treatment challenges. Prog Neuro-Psychopharmacol Biol Psychiatry. (2019) 88:265–75. doi: 10.1016/j.pnpbp.2018.08.001, PMID: 30092241 PMC6459196

[19] Campbell ML Morrison AP. The psychological consequences of combat exposure: the importance of appraisals and post-traumatic stress disorder symptomatology in the occurrence of delusional-like ideas. Br J Clin Psychol. (2007) 46:187–201. doi: 10.1348/014466506X128287, PMID: 17524212

[20] Buck B Norr A Katz A Gahm GA Reger GM. Reductions in reported persecutory ideation and psychotic-like experiences during exposure therapy for posttraumatic stress disorder. Psychiatry Res. (2019) 272:190–5. doi: 10.1016/j.psychres.2018.12.022 30584951

[21] Spencer AE Faraone SV Bogucki OE Pope AL Uchida M Milad MR. Examining the association between posttraumatic stress disorder and attention-deficit/hyperactivity disorder: a systematic review and meta-analysis. J Clin Psychiatry. (2016) 77:72–83. doi: 10.4088/JCP.14r09479 26114394

[22] Wendt FR Garcia-Argibay M Cabrera-Mendoza B Valdimarsdottir UA Gelernter J Stein MB. The relationship of attention-deficit/hyperactivity disorder with posttraumatic stress disorder: a two-sample Mendelian randomization and population-based sibling comparison study. Biol Psychiatry. (2023) 93:362–9. doi: 10.1016/j.biopsych.2022.08.012, PMID: 36335070 PMC10496427

[23] Zhu J Zhang X Xu Y Spencer TJ Biederman J Bhide PG. Prenatal nicotine exposure mouse model showing hyperactivity, reduced cingulate cortex volume, reduced dopamine turnover, and responsiveness to oral methylphenidate treatment. J Neurosci. (2012) 32:9410–8. doi: 10.1523/JNEUROSCI.1041-12.2012, PMID: 22764249 PMC3417040

[24] Eppolito AK Bachus SE McDonald CG Meador-Woodruff JH Smith RF. Late emerging effects of prenatal and early postnatal nicotine exposure on the cholinergic system and anxiety-like behavior. Neurotoxicol Teratol. (2010) 32:336–45. doi: 10.1016/j.ntt.2009.12.009, PMID: 20060465

[25] Milad MR Pitman RK Ellis CB Gold AL Shin LM Lasko NB. Neurobiological basis of failure to recall extinction memory in posttraumatic stress disorder. Biol Psychiatry. (2009) 66:1075–82. doi: 10.1016/j.biopsych.2009.06.026, PMID: 19748076 PMC2787650

[26] Jager A Kanters D Geers F Buitelaar JK Kozicz T Glennon JC. Methylphenidate dose-dependently affects aggression and improves fear extinction and anxiety in BALB/cJ mice. Front Psych. (2019) 10:768. doi: 10.3389/fpsyt.2019.00768, PMID: 31708820 PMC6823535

[27] McAllister TW Zafonte R Jain S Flashman LA George MS Grant GA. Randomized placebo-controlled trial of methylphenidate or Galantamine for persistent emotional and cognitive symptoms associated with PTSD and/or traumatic brain injury. Neuropsychopharmacology. (2016) 41:1191–8. doi: 10.1038/npp.2015.282, PMID: 26361060 PMC4793116

[28] Luderer M Reinhard I Richter A Kiefer F Weber T. ADHD is associated with a higher risk for traumatic events, self-reported PTSD, and a higher severity of PTSD symptoms in alcohol-dependent patients. Eur Addict Res. (2020) 26:245–53. doi: 10.1159/000508918, PMID: 32653887

[29] Antshel KM Biederman J Spencer TJ Faraone SV. The neuropsychological profile of comorbid post-traumatic stress disorder in adult ADHD. J Atten Disord. (2016) 20:1047–55. doi: 10.1177/1087054714522512, PMID: 24567364

[30] Daud A Rydelius PA. Comorbidity/overlapping between ADHD and PTSD in relation to IQ among children of traumatized/non-traumatized parents. J Atten Disord. (2009) 13:188–96. doi: 10.1177/1087054708326271, PMID: 19395643

[31] Biederman J Petty CR Spencer TJ Woodworth KY Bhide P Zhu J. Examining the nature of the comorbidity between pediatric attention deficit/hyperactivity disorder and post-traumatic stress disorder. Acta Psychiatr Scand. (2013) 128:78–87. doi: 10.1111/acps.12011, PMID: 22985097 PMC3527641

[32] Misiak B Szewczuk-Boguslawska M Samochowiec J Moustafa AA Gawęda Ł. Unraveling the complexity of associations between a history of childhood trauma, psychotic-like experiences, depression and non-suicidal self-injury: a network analysis. J Affect Disord. (2023) 337:11–7. doi: 10.1016/j.jad.2023.05.044, PMID: 37230261

[33] Misiak B Samochowiec J Gaweda L Frydecka D. Association of sociodemographic, proximal, and distal clinical factors with current suicidal ideation: findings from a nonclinical sample of young adults. Eur Psychiatry. (2023) 66:e29. doi: 10.1192/j.eurpsy.2023.14, PMID: 36847110 PMC10044310

[34] Misiak B Frydecka D Kowalski K Samochowiec J Jablonski M Gaweda L. Associations of neurodevelopmental risk factors with psychosis proneness: findings from a non-clinical sample of young adults. Compr Psychiatry. (2023) 123:152385. doi: 10.1016/j.comppsych.2023.152385, PMID: 36931184

[35] Gaweda L Kokoszka A. Polish version of the revised hallucination scale (RHS) by Morrison Kokoszka a its factor analysis and the prevalence of hallucinatory-like experiences among healthy participants. Psychiatr Pol. (2011) 45:527–43. PMID: 22232979

[36] Morrison AP Wells A Nothard S. Cognitive and emotional predictors of predisposition to hallucinations in non-patients. Br J Clin Psychol. (2002) 41:259–70. doi: 10.1348/014466502760379127, PMID: 12396254

[37] Morrison AP Wells A Nothard S. Cognitive factors in predisposition to auditory and visual hallucinations. Br J Clin Psychol. (2000) 39:67–78. doi: 10.1348/014466500163112 10789029

[38] Freeman D Loe BS Kingdon D Startup H Molodynski A Rosebrock L. The revised Green et al., paranoid thoughts scale (R-GPTS): psychometric properties, severity ranges, and clinical cut-offs. Psychol Med. (2021) 51:244–53. doi: 10.1017/S0033291719003155, PMID: 31744588 PMC7893506

[39] Ising HK Veling W Loewy RL Rietveld MW Rietdijk J Dragt S. The validity of the 16-item version of the prodromal questionnaire (PQ-16) to screen for ultra high risk of developing psychosis in the general help-seeking population. Schizophr Bull. (2012) 38:1288–96. doi: 10.1093/schbul/sbs068, PMID: 22516147 PMC3713086

[40] Hinterbuchinger B Mossaheb N. Psychotic-like experiences: a challenge in definition and assessment. Front Psych. (2021) 12:582392. doi: 10.3389/fpsyt.2021.582392, PMID: 33854445 PMC8039445

[41] Kessler RC Adler L Ames M Demler O Faraone S Hiripi E. The World Health Organization adult ADHD self-report scale (ASRS): a short screening scale for use in the general population. Psychol Med. (2005) 35:245–56. doi: 10.1017/S0033291704002892, PMID: 15841682

[42] Sheehan DV Lecrubier Y Sheehan KH Amorim P Janavs J Weiller E. The Mini-international neuropsychiatric interview (M.I.N.I.): the development and validation of a structured diagnostic psychiatric interview for DSM-IV and ICD-10. J Clin Psychiatry. (1998) 59:22–33. 9881538

[43] Kroenke K Spitzer RL Williams JB. The PHQ-9: validity of a brief depression severity measure. J Gen Intern Med. (2001) 16:606–13. doi: 10.1046/j.1525-1497.2001.016009606.x, PMID: 11556941 PMC1495268

[44] Hennig T Jaya ES Lincoln TM. Bullying mediates between attention-deficit/hyperactivity disorder in childhood and psychotic experiences in early adolescence. Schizophr Bull. (2017) 43:1036–44. doi: 10.1093/schbul/sbw139, PMID: 27803356 PMC5581899

[45] Gracie A Freeman D Green S Garety PA Kuipers E Hardy A. The association between traumatic experience, paranoia and hallucinations: a test of the predictions of psychological models. Acta Psychiatr Scand. (2007) 116:280–9. doi: 10.1111/j.1600-0447.2007.01011.x, PMID: 17803758

[46] Lovatt A Mason O Brett C Peters E. Psychotic-like experiences, appraisals, and trauma. J Nerv Ment Dis. (2010) 198:813–9. doi: 10.1097/NMD.0b013e3181f97c3d, PMID: 21048472

[47] Freeman D Fowler D. Routes to psychotic symptoms: trauma, anxiety and psychosis-like experiences. Psychiatry Res. (2009) 169:107–12. doi: 10.1016/j.psychres.2008.07.009, PMID: 19700201 PMC2748122

[48] Cuffe SP McCullough EL Pumariega AJ. Comorbidity of attention deficit hyperactivity disorder and post-traumatic stress disorder. J Child Fam Stud. (1994) 3:327–36. doi: 10.1007/BF02234689

[49] Iacono WG Malone SM McGue M. Behavioral disinhibition and the development of early-onset addiction: common and specific influences. Annu Rev Clin Psychol. (2008) 4:325–48. doi: 10.1146/annurev.clinpsy.4.022007.141157, PMID: 18370620

[50] Degenhardt L Coffey C Hearps S Kinner SA Borschmann R Moran P. Associations between psychotic symptoms and substance use in young offenders. Drug Alcohol Rev. (2015) 34:673–82. doi: 10.1111/dar.12280, PMID: 26084677

[51] Fonseca-Pedrero E Ortuno-Sierra J Paino M Muniz J. Psychotic-like experiences and substance use in college students. Adicciones. (2016) 28:144–53. doi: 10.20882/adicciones.781 27399223

[52] Wimberley T Agerbo E Horsdal HT Ottosen C Brikell I Als TD. Genetic liability to ADHD and substance use disorders in individuals with ADHD. Addiction. (2020) 115:1368–77. doi: 10.1111/add.14910, PMID: 31803957

[53] Lipschitz DS Grilo CM Fehon D McGlashan TM Southwick SM. Gender differences in the associations between posttraumatic stress symptoms and problematic substance use in psychiatric inpatient adolescents. J Nerv Ment Dis. (2000) 188:349–56. doi: 10.1097/00005053-200006000-00005, PMID: 10890343

[54] Khoury L Tang YL Bradley B Cubells JF Ressler KJ. Substance use, childhood traumatic experience, and posttraumatic stress disorder in an urban civilian population. Depress Anxiety. (2010) 27:1077–86. doi: 10.1002/da.20751, PMID: 21049532 PMC3051362

[55] Reynolds M Mezey G Chapman M Wheeler M Drummond C Baldacchino A. Co-morbid post-traumatic stress disorder in a substance misusing clinical population. Drug Alcohol Depend. (2005) 77:251–8. doi: 10.1016/j.drugalcdep.2004.08.017, PMID: 15734225

[56] Wright KB. Researching internet-based populations: advantages and disadvantages of online survey research, online questionnaire authoring software packages, and web survey services. J Comput-Mediat Comm. (2005) 10. doi: 10.1111/j.1083-6101.2005.tb00259.x

[57] Bak M Delespaul P Hanssen M de Graaf R Vollebergh W van Os J. How false are “false” positive psychotic symptoms? Schizophr Res. (2003) 62:187–9. doi: 10.1016/S0920-9964(02)00336-5 12765760

[58] van der Steen Y Myin-Germeys I van Nierop M Ten Have M de Graaf R van Dorsselaer S. ‘False-positive’ self-reported psychotic experiences in the general population: an investigation of outcome, predictive factors and clinical relevance. Epidemiol Psychiatr Sci. (2019) 28:532–43. doi: 10.1017/S2045796018000197, PMID: 29656729 PMC6998918

